# Clinicopathological Features Combined With Immune Infiltration Could Well Distinguish Outcomes in Stage II and Stage III Colorectal Cancer: A Retrospective Study

**DOI:** 10.3389/fonc.2021.776997

**Published:** 2021-12-03

**Authors:** Jiazi Ren, Linfeng Xu, Siyu Zhou, Jian Ouyang, Weiqiang You, Nengquan Sheng, Li Yan, Du Peng, Lu Xie, Zhigang Wang

**Affiliations:** ^1^ Department of General Surgery, Shanghai Jiao Tong University Affiliated Sixth People’s Hospital, Shanghai, China; ^2^ Shanghai Center for Bioinformation Technology, Shanghai Institute for Biomedical and Pharmaceutical Technologies, Shanghai, China; ^3^ School of Health Science and Engineering, University of Shanghai for Science and Technology, Shanghai, China; ^4^ Department of Colorectal Surgery, Xinhua Hospital, Shanghai Jiao Tong University School of Medicine, Shanghai, China

**Keywords:** clinicopathological features, Immunoscore, colorectal cancer, prognostic, model

## Abstract

**Background:**

The Immunoscore predicts prognosis in patients with colorectal cancer (CRC). However, a few studies have incorporated the Immunoscore into the construction of comprehensive prognostic models in CRC, especially stage II CRC. We aimed to construct and validate multidimensional models integrating clinicopathological characteristics and the Immunoscore to predict the prognosis of patients with stage II–III CRC.

**Methods:**

Patients (*n* = 254) diagnosed with stage II–III CRC from 2009 to 2016 were used to generate Cox models for predicting disease-free survival (DFS) and overall survival (OS). The variables included basic clinical indicators, blood inflammatory markers, preoperative tumor biomarkers, mismatch repair status, and the Immunoscore (CD3^+^ and CD8^+^ T-cell densities). Univariate and multivariate Cox proportional regressions were used to construct the prognostic models for DFS and OS. We validated the predictive accuracy and ability of the prognostic models in our cohort of 254 patients.

**Results:**

We constructed two predictive prognostic models with C-index values of 0.6941 for DFS and 0.7138 for OS in patients with stage II–III CRC. The Immunoscore was the most informative predictor of DFS (11.92%), followed by pN stage, carcinoembryonic antigen (CEA), and vascular infiltration. For OS, the Immunoscore was the most informative predictor (8.59%), followed by pN stage, age, CA125, and CEA. Based on the prognostic models, nomograms were developed to predict the 3- and 5-year DFS and OS rates. Patients were divided into three risk groups (low, intermediate, and high) according to the risk scores obtained from the nomogram, and significant differences were observed in the recurrence and survival of the different risk groups (*p* < 0.0001). Calibration curve and time-dependent receiver operating characteristic (ROC) analysis showed good accuracy of our models. Furthermore, the decision curve analysis indicated that our nomograms had better net benefit than pathological TNM (pTNM) stage within a wide threshold probability. Especially, we developed a website based on our prognostic models to predict the risks of recurrence and death of patients with stage II–III CRC.

**Conclusions:**

Multidimensional models including the clinicopathological characteristics and the Immunoscore were constructed and validated, with good accuracy and convenience, to evaluate the risks of recurrence and death of stage II–III CRC patients.

## Introduction

Colorectal cancer (CRC) is a highly prevalent and dangerous global disease. In 2020, CRC ranked the third (10%) in morbidity and the second (9.4%) in mortality in the world (1). The morbidity and mortality of CRC in China ranked the second (12.2%) and the fifth (9.5%) (1), respectively. Despite improvements in diagnosis and treatment technology, 30% of stage II–III CRC patients still suffer recurrence after radical surgery (2), which seriously affects patient prognosis. Currently, the pathological TNM (pTNM) staging system based on the 8th edition of the American Joint Committee on Cancer (AJCC) is the main standard in prognostic evaluation, adjuvant treatment, and follow-up strategy in patients after curative CRC surgery (3). However, in clinical practice, discordances are usually observed between pTNM stage-based predictions and the actual outcomes. For example, some patients with stage II CRC have worse prognosis than some patients with stage III CRC (4, 5). Therefore, the prognostic information provided by the current evaluation system is limited. The major reasons for this include the lack of immune infiltration, tumor genetic status, and DNA mismatch repair (MMR) status, among others. Although some researchers have explored new indicators to improve prognosis prediction (6, 7), their clinical application is still very limited. Accordingly, it is of great importance and urgent clinical significance to explore approaches with good clinical feasibility and accuracy to predict the risks of recurrence and death of CRC patients.

In colon cancer, Galon et al. proposed the Immunoscore concept (8), which is a quantification of CD3^+^ and CD8^+^ T cells in the tumor core (CT) and invasive margin (IM). A multicenter international collaboration group verified the prognostic value of the Immunoscore in stage I–III colon cancer, and the relative contribution of Immunoscore was the largest among all risk factors, even more than that of the pTNM staging system. Notably, the combination of the Immunoscore and clinical indicators significantly improved the predictive accuracy for overall survival (9). These studies suggest that the Immunoscore could be a powerful complement to the existing prognostic evaluation systems.

In addition to the Immunoscore, the tumor location characteristics (10, 11), molecular characteristics (12–14), preoperative tumor markers (15, 16), and tumor inflammatory status (17) are all closely related to CRC prognosis. However, the specificity and the accuracy of the various risk factors are low when used in isolation, making it difficult to accurately assess the prognosis of CRC, whereas the integration of multiple factors into one model will greatly improve the prognostic value (6). Therefore, the construction of a comprehensive CRC prognostic model would be beneficial in improving the accuracy of prognosis prediction. Currently, some groups have used this idea to construct prognostic models for stage III colon cancer (18, 19). However, a comprehensive prognostic model for stage II CRC still remains to be explored.

To solve the above issues, we integrated 18 variables, including the basic clinical indicators, preoperative serum tumor markers, blood inflammatory markers, MMR status, and the Immunoscore, and used the Cox risk proportion model to build new multidimensional models for predicting the recurrence and survival in patients with stage II–III CRC. Our study generated accurate and feasible approaches to prognosis prediction for patients with stage II–III CRC, providing new insights into improving the current prognostic evaluation system and the quality of decision-making for postoperative follow-up and adjuvant treatment.

## Materials and Methods

### Study Population and Data Collection

This study was a single-center retrospective study registered in the Chinese Clinical Trial Registry (approval no. ChiCTR2000041147). Our study was approved by the Ethics Committee of Shanghai Jiao Tong University Affiliated Sixth People’s Hospital (approval no. 2020-253). The cohort from Shanghai Jiao Tong University Affiliated Sixth People’s Hospital was used to develop and validate the model. All patients were pathologically diagnosed with stage II–III CRC between January 2009 and December 2016. Written informed consent was obtained for this study. Patients fulfilling the criteria patients were excluded: 1) age <18 years; 2) had emergency surgery; 3) had multiple primary carcinoma; 4) with incomplete clinical data; 5) died within 30 days; 6) lost to follow-up; and 7) underwent preoperative adjuvant therapy.

In our study, clinical features such as gender, age, pTNM stage, tumor location, tumor cross-sectional area (CSA), tumor long axis, tumor differentiation, lymphatic infiltration, vascular infiltration, nerve infiltration, neutrophil-to-lymphocyte ratio (NLR), platelet-to-lymphocyte ratio (PLR), preoperative tumor markers [carcinoembryonic antigen (CEA), carbohydrate antigen 19-9 (CA19-9), and CA125], and the MMR status were collected. Patient clinical data were mainly provided by examination of their medical history and by the Electronic Medical Record Department. Pathological staging was based on the 8th AJCC criterion for CRC. NLR and PLR were calculated as (neutrophil count)/(lymphocyte count) and (platelet count)/(lymphocyte count), respectively. Preoperative tumor markers were examined within 1 week before surgery. All patients were followed up according to the current National Comprehensive Cancer Network (NCCN) guidelines, including analysis of serum tumor markers, colonoscopy, chest X-ray, and CT (or MRI). Patient follow-up data were updated by telephone, email, and medical history. Disease-free survival (DFS) was defined as the time from surgery to cancer metastasis or recurrence. Overall survival (OS) was defined as the time from surgery to death.

### Immunohistochemical Analysis

Immunostaining of CD3^+^ and CD8^+^ T cells was performed on formalin-fixed paraffin-embedded sections. Antigen retrieval was conducted with an EDTA buffer (pH 9.0) for 90 s, followed by quenching of endogenous peroxidase activity by 3% H_2_O_2_ for 30 min at room temperature. Sections were incubated at 4°C with primary antibodies: rabbit anti-human monoclonal antibody against CD3 (EP41; ZSGB-BIO, Beijing, China) and rabbit anti-human monoclonal antibody against CD8 (SP16; ZSGB-BIO, Beijing, China). Revelation with the Ultra DAB IHC Detection Kit (Maxim, Fuzhou, China) and counterstaining with Harris hematoxylin were performed. Counterstained slides were scanned at ×40 magnification (NanoZoomer S360, Hamamatsu, Japan) to generate a whole slide imaging file in NDPI format. CT was the core of the tumor, and the invasive margin (IM) was defined as a region of 500-μm width surrounding the CT. The CT and IM regions were manually marked on the whole slide using QuPath software (20), in which hematoxylin/eosin-stained sections were used to help CT/IM labeling. Two independent pathologists, who were blinded to the patients’ clinical information, participated in the analysis to avoid the interference of necrotic areas and to verify the location of the CT/IM. Positive CD3 and CD8 cells within the CT and IM areas were obtained *via* QuPath software (20), and the densities of CD3 and CD8 were quantified by the number of cells per square millimeter in both CT and IM. The concordance in the semi-quantitative evaluation between CD3 and CD8 was determined by two independent pathologists.

For every patient, the densities of CD3^+^ and CD8^+^ cells in the CT and IM regions (CD3_CT_, CD3_IM_, CD8_CT_, and CD8_IM_) were converted into percentiles (0%–100%) based on our cohort, as described by Galon et al. (9). The mean of the four percentiles (CD3_CT_, CD3_IM_, CD8_CT_, and CD8_IM_) was then calculated and converted into a percentile Immunoscore. In a three-category Immunoscore analysis, a 0%–25% density was scored as low, 25%–70% density was scored as intermediate, and 70%–100% density was scored as high (9). In our study, we found that a low (0%–25%) and an intermediate (25%–70%) Immunoscore in the three-category Immunoscore had similar clinical outcomes (DFS). Consequently, we combined the low-Immunoscore (0%–25%) and intermediate-Immunoscore (25%–70%) groups as the low-Immunoscore group (0%–70%) in a two-category Immunoscore, in which a 0%–70% density was scored as low and 70%–100% was scored as high. Samples were excluded from the analysis if counts were missing from a tumor region, if there was improper histology (e.g., broken tissue, atypical CT/IM, excessive necrotic cavity, excessive mucous area, etc.), or if the staining intensity was regarded as low.

The tumor DNA MMR status was determined by immunohistochemical analysis of MMR proteins (MLH1, MSH2, MSH6, and PMS2) on formalin-fixed paraffin-embedded sections. The conditions of having deficient MMR (dMMR; loss of at least one MMR protein) and proficient MMR (pMMR) were denoted as microsatellite instability (MSI) and microsatellite stability (MSS), respectively. Two independent pathologists, who were blinded to the patients’ clinical information, participated in the analysis to verify the MMR status.

### Statistical Analysis

The association between the clinicopathological characteristics and the Immunoscore was analyzed *via* a chi-squared test. All numeric variables were tested for normality using the Shapiro–Wilk test.

To develop the prognostic model, we performed univariate analysis of all variables using Cox proportional hazards regression. Subsequently, the significant variables (*p* < 0.05) were analyzed with the multivariate Cox proportional hazards regression. After removing the non-significant covariates in the multivariate analysis, a final multivariable Cox regression model was constructed. A nomogram was constructed to predict the 3- and 5-year DFS/OS probabilities with the total points of all variables. The risk score was the linear predictor of the Cox model built on our cohort with selected variables. To evaluate the predictive accuracy of the different variables or models, we used the integrated area under the receiver operating characteristic (ROC) curve (iAUC) with 1,000× bootstrap resampling. The performances of the models were compared using likelihood ratio tests, when the models were nested. The relative importance of each variable to the risks of recurrence and death was estimated using the *χ* ^2^ from Harrell’s rms R package (version 6.0-1).

Model performance was evaluated with the concordance index (C-index) and corrected 1,000 times by bootstrapping. The calibration curves of the nomogram were drawn for 3- and 5-year DFS/OS to evaluate the accuracy of the model by comparing the DFS/OS probabilities between observations and predictions. A time-dependent ROC was used to compare the discrimination between our nomogram and pTNM. Patients were classified into three risk groups (high, intermediate, and low) according to the risk scores obtained from the nomogram: the 30% with the highest scores were designated the “high” risk group, the 30% with the lowest scores the “low” group, and the remaining 40% as the “intermediate” group. The Kaplan–Meier (K-M) method was applied to estimate the survival probabilities. Hazard ratios (HRs) and 95% confidence intervals (CIs) were estimated, and a log-rank test was used to determine the statistical differences between different groups. Decision curve analysis was conducted using the ggDCA package in R (version 1.2) to determine the clinical usefulness of the nomogram *via* quantifying the net benefits at different threshold probabilities (21).

Data processing, data analysis, and figures were performed and produced in R language (version 4.0.3). All analyses were two sided, and *p* < 0.05 was considered statistically significant.

## Results

### Study Design and Patient Characteristics

A total of 1,048 patients with stage II–III CRC were collected between January 2009 and December 2016 from Shanghai Jiao Tong University Affiliated Sixth People’s Hospital (**Supplementary Figure S1**). After clinical quality control, there were 735 eligible patients with complete clinical data. Formalin-fixed paraffin-embedded sections of tumor samples from 350 out of the 735 patients were collected and the Immunoscore data were retrieved between 2020 and 2021. Within the 350 samples, 96 were excluded due to mismatch in the quality control, among which 67 patients were excluded after histology quality control, 20 patients were excluded after staining quality control, and 9 patients were excluded due to missing staining data. Subsequently, only eligible patients with qualified immunohistochemical data samples (*n* = 254) were finally included in the development and validation of the prognostic models.

The characteristics of our study population are shown in **Table 1**. In total, 60.0% of patients were males, and the median age of all patients was 66.0 years (IQR = 56–76 years). One hundred fifty-one (59.0%) patients had stage II and 103 (41.0%) had stage III CRC. Colon tumors located on the left and right sides were 73 (29.0%) and 84 (33.0%), respectively, and 97 (38.0%) patients had rectum tumors. The degree of differentiation in more than half of the tumors was identified as moderate or well (147, 58.0%), and 107 (42.0%) patients had a poor level. Of the patients, 235 (93.0%) showed MSS and only 19 (7.0%) showed MSI. Seventy-seven (30.0%) patients had a relapse, and 74 (29.0%) patients died. The median follow-up time for all patients was 53.0 months.

**Table 1:** Clinicopathological and molecular characteristics of patients with stage II–III colorectal cancer (CRC).

Variable	Patients (*n* = 254)
Gender, *n* (%)	
Female	101 (40.0)
Male	153 (60.0)
Age	
Median	66.0
Interquartile range	56.0–76.0
pT stage, *n* (%)	
T1, T2, T3	61 (24.0)
T4	193 (76.0)
pN stage, *n* (%)	
N0	151 (59.0)
N1	67 (26.0)
N2	36 (14.0)
pTNM stage, *n* (%)	
II	151 (59.0)
III	103 (41.0)
Tumor location, *n* (%)	
Left colon	73 (29.0)
Right colon	84 (33.0)
Rectum	97 (38.0)
Tumor CSA, *n* (%)	
<16	123 (48.0)
≥16	131 (52.0)
Tumor long axis, *n* (%)	
<4.5	124 (49.0)
≥4.5	130 (51.0)
Degree of tumor differentiation, *n* (%)	
Moderate and well	147 (58.0)
Poor	107 (42.0)
Lymphatic infiltration, *n* (%)	
Absent	108 (43.0)
Present	146 (57.0)
Vascular infiltration, *n* (%)	
Absent	224 (88.0)
Present	30 (12.0)
Nerve infiltration, *n* (%)	
Absent	14 (6.0)
Present	240 (94.0)
NLR	
Median	2.2
Interquartile range	1.6–3.5
PLR	
Median	7.3
Interquartile range	103.0–216.1
CEA	
Median	3.9
Interquartile range	2.0–9.5
CA19-9	
Median	12.0
Interquartile range	6.8–24.1
CA125	
Median	10.8
Interquartile range	8.3–16.7
MMR, *n* (%)	
dMMR	19 (7.0)
pMMR	235 (93.0)
Immunoscore, *n* (%)	
Low	189 (74.0)
High	65 (26.0)
Metastasis or recurrence, *n* (%)	
No	177 (70.0)
Yes	77 (30.0)
Survival status, *n* (%)	
Alive	180 (71.0)
Dead	74 (29.0)

CSA, tumor cross-sectional area; NLR, neutrophil-to-lymphocyte ratio; PLR, platelet-to-lymphocyte ratio; dMMR, deficient mismatch repair; pMMR, proficient mismatch repair.

The median densities of CD3^+^ T cells in CT and IM were 227/mm^2^ (14–2,061/mm^2^) and 629/mm^2^ (38–2,359/mm^2^), respectively, and those of CD8^+^ T cells were 119/mm^2^ (8–1,832/mm^2^) and 340/mm^2^ (13–1,365/mm^2^), respectively. More than half of CRC patients had a low Immunoscore (189, 74.0%), while 26.0% (65) of patients had a high Immunoscore (**Table 1**).

To study the association between the Immunoscore and other characteristics in the tumor microenvironment, we performed chi-squared test analysis (**Supplementary Table S1**). We did not find any relationship between the Immunoscore and other characteristics, except for the microsatellite status. The results showed that a high Immunoscore was found more frequently than a low Immunoscore in tumors with dMMR (14.0% *vs*. 5.0%, *p* = 0.0468). Although tumor location was not significantly associated with the Immunoscore (*p* = 0.0689), it showed some trends, and a high Immunoscore was found to be less frequent in tumors in right-sided colon cancer (**Supplementary Table S1**).

### Validation of the Two-Level Categorical Immunoscore for Predicting DFS and OS

**Figure 1:**
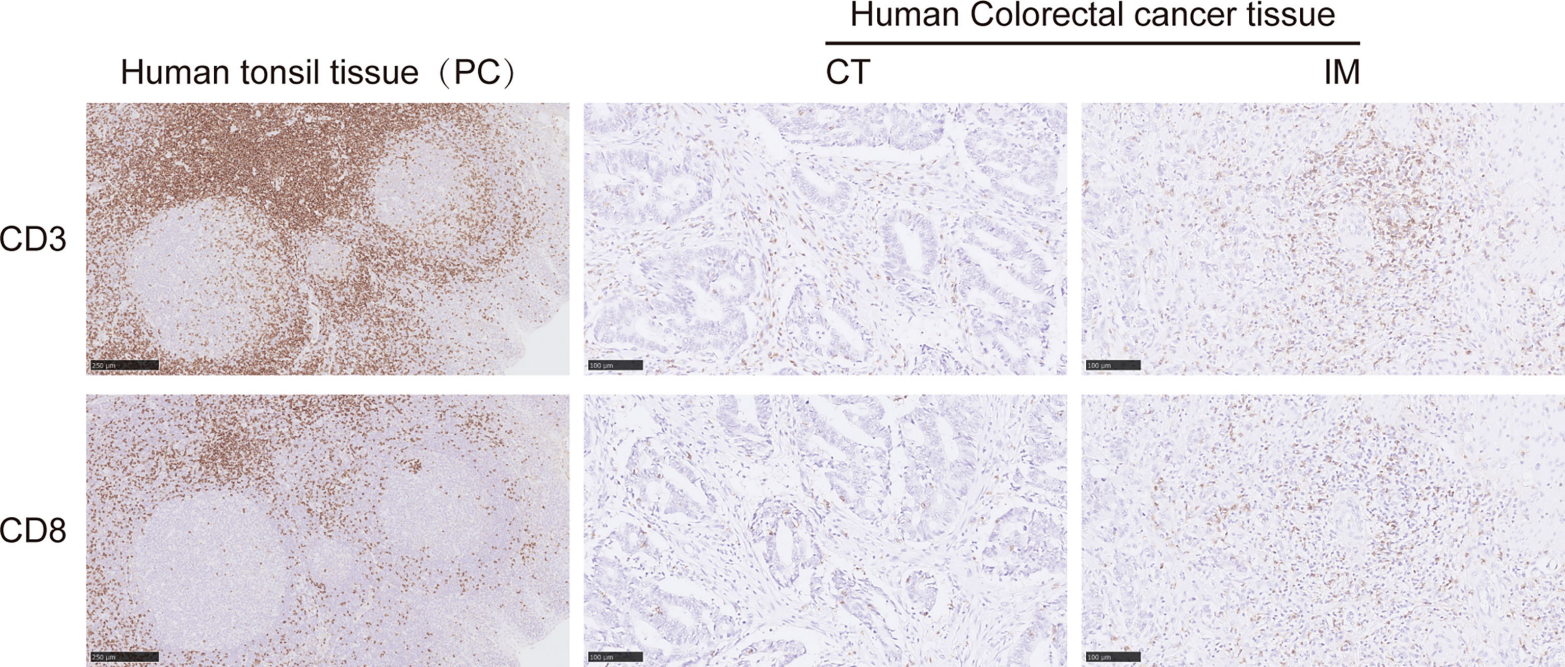
Representative immunohistochemical images of CD3^+^ and CD8^+^ T cells on formalin-fixed paraffin-embedded sections from human colorectal cancer (CRC) tissue. Representative images of CD3^+^ T cells in the CT (*middle upper panel*) and IM (*right upper panel*) and CD8^+^ T cells in the CT (*middle lower panel*) and IM (*right lower panel*). Human tonsil tissues were stained as positive controls for CD3 and CD8 positive staining (*left upper* and *lower panels*). CT, the core of the tumor; IM, invasive margin.

Representative images of CD3^+^ and CD8^+^ T-lymphocyte immunostaining on formalin-fixed paraffin-embedded sections are provided in **Figure 1**. The CT and IM areas of the tumor were manually marked on the whole slide using QuPath software (20), in which hematoxylin/eosin-stained sections were used to help in CT/IM labeling. We validated the two-level categorical Immunoscore (**Supplementary Figure S2**) whose prognostic impact was previously shown in an international validation study in TNM stage I–III colon cancers (9). When tumors were categorized into predetermined low (0%–70%) and high (70%–100%) groups, a low Immunoscore was associated with a statistically significant and poorer DFS (*p* = 0.0390) and OS (*p* = 0.0070). The 3-year DFS for low *vs*. high Immunoscore was 68.7% *vs*. 82.5%, and the 3-year OS was 75.9% *vs*. 87.4%. The 5-year DFS for low *vs*. high Immunoscore was 62.6% *vs*. 77.3%, and the 5-year OS was 63.1% *vs*. 82.1% (**Supplementary Figure S2**). For consistency with prior work, the associations between DFS and OS were also shown for percentile Immunoscore and three-level categorical Immunoscore (9) (**Supplementary Table S2**).

### Association of Clinicopathological Variables and Immunoscore With DFS and OS

To screen the prognostic factors, we performed univariate analysis. Our results suggested that the pT stage, pN stage, lymphatic infiltration, CEA, and the Immunoscore were significantly associated with DFS and OS in CRC patients (*p* < 0.05) (**Supplementary Table S2**). Vascular infiltration affected DFS, but not OS, whereas age, NLR, and CA125 affected OS, but not DFS (*p* < 0.05) (**Supplementary Table S2**).

Compared to patients with T1, T2, and T3 tumors, patients with T4 tumors had significantly worse DFS (HR = 2.05, 95% CI = 1.11–3.80, *p* = 0.0226) and OS (HR = 2.58, 95% CI = 1.32–5.04, *p* = 0.0054) (**Supplementary Table S2**). Patients with N2 had statistically significant and worse DFS (HR = 3.98, 95% CI = 2.34–6.78, *p* < 0.0001) and OS (HR = 4.27, 95% CI = 2.47–7.37, *p* < 0.0001) than patients with N0. Patients with N1 also had worse OS (HR = 2.05, 95% CI = 1.11–3.80, *p* = 0.0226), but not DFS, than patients with N0 (**Supplementary Table S2**). Patients with lymphatic infiltration displayed a statistically significant association with DFS (*p* < 0.05) and OS (*p* < 0.05) (**Supplementary Table S2**) and were therefore included in the multivariable Cox models. An increased level of CEA was associated with shorter DFS (*p* < 0.0001) and OS (*p* < 0.01), which achieved statistical significance (**Supplementary Table S2**). In the univariate analysis, the Immunoscore was analyzed as a two-level categorical variable, and a high Immunoscore was associated with a statistically significant and better DFS (HR = 0.54, 95% CI = 0.30–0.98, *p* = 0.0421) and OS (HR = 0.41, 95% CI = 0.21–0.80, *p* = 0.0092) (**Supplementary Table S2**). For consistency with prior studies, the associations with DFS and OS were also shown for the percentile Immunoscore and three-level categorical Immunoscore (9), and both were protective prognostic factors for DFS (*p* < 0.05) and OS (*p* < 0.05) (**Supplementary Table S2**). The MMR status was not prognostic either in DFS (*p* = 0.5753) or OS (*p* = 0.3085) (**Supplementary Table S2**). Subsequently, all variables statistically significant in the univariate analysis were entered into the multivariate Cox proportional hazards regression analysis.

### Construction of Prognostic Models Using Multivariate Cox Proportional Hazards Regression Analyses

Multivariate Cox proportional hazards regression analysis for DFS and OS revealed that the pN stage, vascular infiltration, CEA, and the Immunoscore were independently associated with DFS (*p* < 0.05) (**Table 2**), and age, pN stage, CEA, CA125, and the Immunoscore were independently associated with OS (*p* < 0.05) (**Table 2**) in our cohort. The predictive accuracy of the Immunoscore was evaluated by determining the time-dependent AUC (**Supplementary Figure S3**). For DFS, the predictive accuracy of the Immunoscore was found to be similar to that of the PLR (*p* > 0.05) and was superior to that of gender, age, tumor location, tumor CSA, tumor long axis, tumor differentiation, nerve infiltration, NLR, CA19-9, CA125, or MMR (*p* < 0.05), however was lower than that of pT stage, pN stage, lymphatic infiltration, vascular infiltration, and CEA (*p* < 0.05) (**Supplementary Figure S3A**). For OS, the predictive accuracy of the Immunoscore was found to be similar to that of pT stage (*p* > 0.05) and was superior to that of gender, age, tumor location, tumor CSA, tumor long axis, tumor differentiation, lymphatic infiltration, vascular infiltration, nerve infiltration, NLR, PLR, CA19-9, or MMR (*p* < 0.05), however was lower than that of pN stage, CEA, and CA125 (*p* < 0.05) (**Supplementary Figure S3B**). Furthermore, adding preoperative serum tumor markers (CEA, CA19-9, and CA125) or the Immunoscore to a model that combined all clinical variables (gender, age, pT stage, pN stage, tumor location, tumor CSA, tumor long axis, tumor differentiation, lymphatic infiltration, vascular infiltration, and nerve infiltration) significantly improved both DFS (likelihood ratio: *p* = 0.0052 and *p* = 0.0276, respectively) and OS (likelihood ratio: *p* = 0.0004 and *p* = 0.0117, respectively) prediction (**Supplementary Figures S3A, B**). Therefore, clinical variables, preoperative serum tumor markers, and the Immunoscore were all required to optimize the determination of patient prognosis.

**Table 2:** Multivariate Cox proportional hazards regression analysis for disease-free survival (DFS) and overall survival (OS).

Variable	Disease-free survival		Overall survival	
	HR (95% CI)	*p*-value	HR (95% CI)	*p*-value
Age			1.96 (1.32–2.91)	0.0009
pT stage	0.65 (0.35–1.24)	0.1934	1.92 (0.96–3.83)	0.0640
pN stage				
N1 *vs*. N0	1.16 (0.66–2.05)	0.6087	1.69 (0.96–2.98)	0.0707
N2 *vs*. N0	3.49 (2.03–6.02)	<0.0001	5.50 (3.05–9.92)	<0.0001
Lymphatic infiltration				
Present *vs*. absent	0.74 (0.38–1.46)	0.3882	0.77 (0.40–1.49)	0.4385
Vascular infiltration				
Present *vs*. absent	2.08 (1.18–3.65)	0.0108		
CEA	1.75 (1.36–2.27)	<0.0001	1.40 (1.05–1.87)	0.0202
CA125			1.41 (1.14–1.73)	0.0012
NLR			1.06 (0.99–1.14)	0.1118
Immunoscore				
High *vs*. low	0.46 (0.25–0.84)	0.0117	0.44 (0.22–0.87)	0.0179
C-index	0.6941		0.7138	

CEA and CA125 are processed by logarithmic transformation (base e).

CI, confidence interval; HR, hazard ratio; CEA, carcinoembryonic antigen; CA125, carbohydrate antigen 125; NLR, neutrophil-to-lymphocyte ratio.

Variables that were statistically significant in the multivariate Cox analysis were used to develop the final prognostic models, which included independent variables that were associated with DFS and OS. Finally, four indicators were selected for the prognostic model of DFS in CRC, including pN stage, vascular infiltration, CEA, and the Immunoscore (**Table 2**), and five indicators were selected for OS prediction in CRC, including age, pN stage, CEA, CA125, and the Immunoscore (**Table 2**).

**Figure 2:**
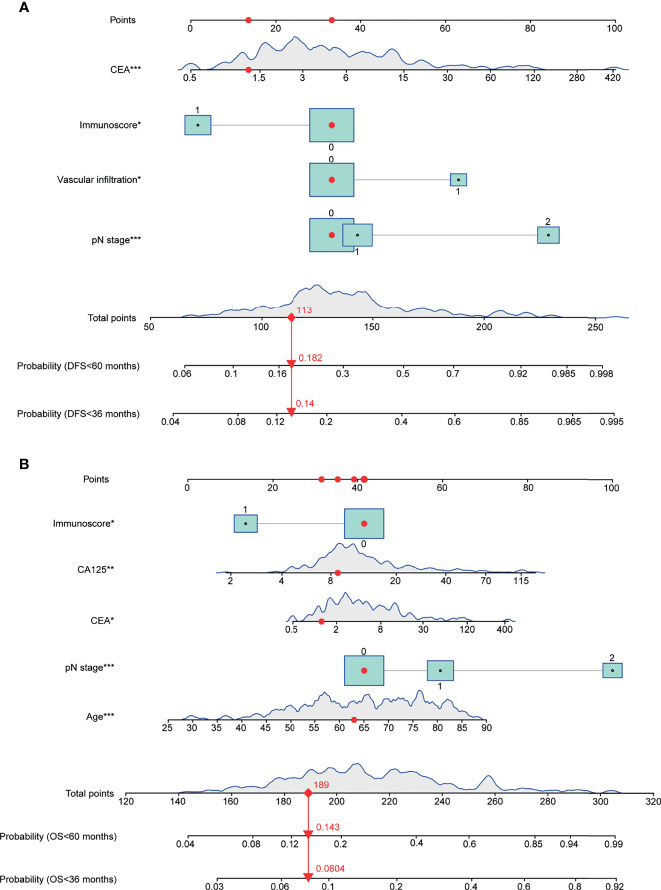
Nomograms for predicting the 3- and 5-year DFS/OS probabilities based on multivariable Cox models. **(A)** Nomogram for DFS prediction. **(B)** Nomogram for OS prediction. Continuous or category variables were shown by *peaks* or *rectangles*, respectively. The distribution of the peak represents the distribution of sample size, and the *size of the rectangles* represents the sample size within each category. The nomograms assign points to each variable, and the 3- and 5-year DFS/OS probabilities were predicted by the total points of all variables. The *red points* and *arrows* give an example for DFS and OS prediction. OS, overall survival; DFS, disease-free survival (*P < 0.05, **P < 0.01, ***P < 0.001).

The final model was then used to generate a nomogram to predict the DFS (**Figure 2A**) and OS (**Figure 2B**) rates for individual patients in clinical practice. The nomogram assigns points to each variable and allows predicting the DFS/OS probabilities at 3 and 5 years using the total points of all variables. The risk scores were generated as a linear predictor of the Cox model and were calculated as follows: 1) risk score for DFS in CRC patients: (0*N0 + 0.1488*N1 + 1.2511*N2) + 0.7320*(vascular infiltration) + 0.3625*ln(CEA value) + −0.7759*(Immunoscore); 2) risk score for OS in CRC patients: 0.0336*age + (0*N0 + 0.5231*N1 + 1.7046*N2) + 0.2192*ln(CEA value) + 0.4896*ln(CA125 value) + −0.8166*(Immunoscore).

**Figure 3:**
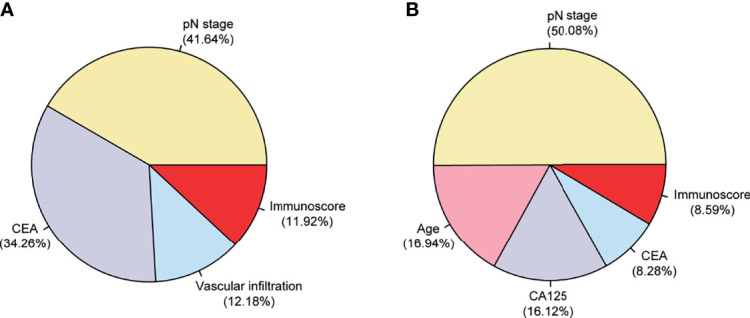
Relative contributions (in percent) of each variable to DFS/OS in the final multivariable Cox models in 254 patients with stage II–III colorectal cancer (CRC). The relative importance of each variable to the risks of DFS **(A)** and OS **(B)**. OS, overall survival; DFS, disease-free survival.

We analyzed the relative importance of all variables in the final multivariable model, and the results revealed that the pN stage (41.64%) had the largest impact on DFS, followed by CEA (34.26%), vascular infiltration (12.18%), and then the Immunoscore (11.92%) (**Figure 3A**). For OS, the pN stage (50.08%) had the largest impact, followed by age (16.94%), CA125 (16.12%), CEA (8.28%), and then the Immunoscore (8.59%) (**Figure 3B**).

### Evaluation and Determination of the Accuracy and Predictive Power of the Prognostic Models

**Figure 4:**
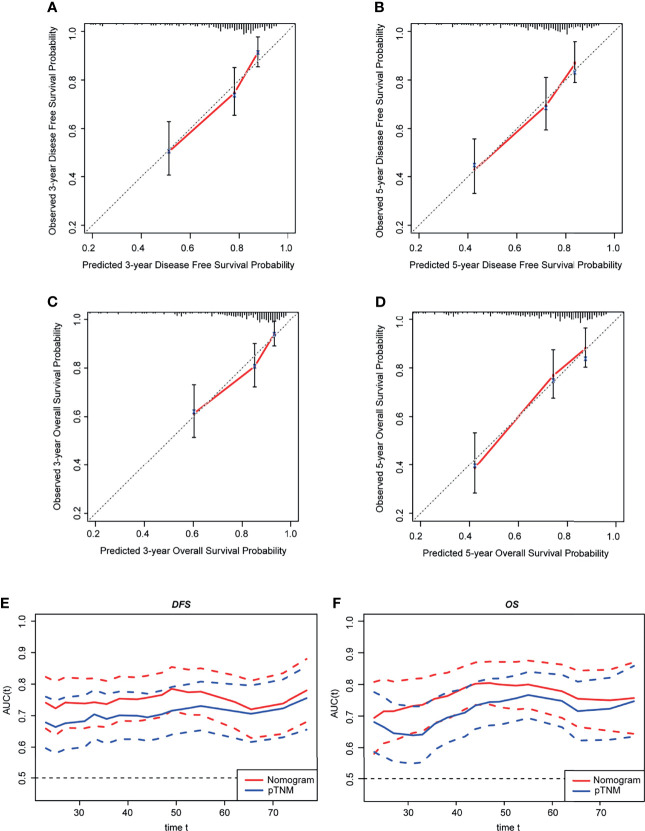
Prognostic accuracy of the nomograms in 254 patients with stage II–III colorectal cancer (CRC). **(A, B)** Calibration curves for 3-year **(A)** and for 5-year **(B)** DFS. **(C, D)** Calibration curves for 3-year **(C)** and for 5-year **(D)** OS. **(E, F)** Time-dependent ROC curves comparing the prognostic accuracy of the nomograms with pTNM stage for DFS **(E)** and OS **(F)**. The *x*-axis in **(A–D)** shows the predicted 3- and 5-year DFS/OS probabilities by the nomogram, and the *y*-axis shows the observed 3- and 5-year DFS/OS probabilities. *Dashed lines* represent perfect prediction (accuracy is 100%), and *red solid lines* represent the actual prediction. ROC, receiver operating characteristic; pTNM, pathological TNM based on the 8th edition American Joint Committee on Cancer (AJCC); OS, overall survival; DFS, disease-free survival.

The C-index of the nomogram was 0.6941, corrected with 1,000 permutations, for DFS in CRC in our cohort (**Table 2**). The C-index of our OS model was 0.7138, corrected with 1,000 permutations (**Table 2**). Notably, the C-index of pTNM based on the 8th edition of AJCC was 0.6456 for DFS and was 0.6647 for OS. The calibration curves for CRC based on the nomograms showed very good agreement between the predicted and observed probabilities of DFS and OS at 3 and 5 years (**Figures 4A–D**). Consistently, our nomogram also showed a slightly higher prognostic accuracy than the pTNM stage from 30 to 70 months for both DFS (3-year AUC: nomogram = 0.74, pTNM = 0.69; 5-year AUC: nomogram = 0.75, pTNM = 0.71) (**Figure 4E**) and OS (3-year AUC: nomogram = 0.75, pTNM = 0.68; 5-year AUC: nomogram = 0.78, pTNM = 0.74) (**Figure 4F**) in the time-dependent ROC analysis.

**Figure 5:**
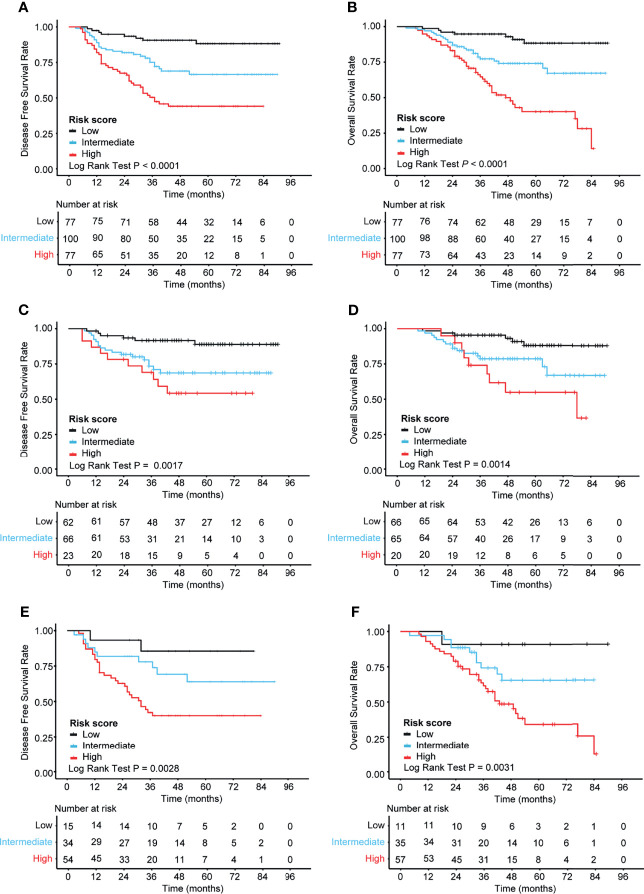
Kaplan–Meier analysis of the different risk groups in 254 patients with stage II–III colorectal cancer (CRC). **(A, B)** K-M curves for DFS **(A)** and OS **(B)** in all patients (*n* = 254). **(C, D)** K-M curves for DFS **(C)** and OS **(D)** in patients with stage II CRC (*n* = 151). **(E, F)** K-M curves for DFS **(E)** and OS **(F)** in patients with stage III CRC (*n* = 103). The log-rank test was performed to determine statistical differences. K-M, Kaplan–Meier; OS, overall survival; DFS, disease-free survival.

According to the risk scores obtained from the nomogram, the patients were categorized into three risk groups: 30% of patients with the highest scores classified as “high”, 30% with the lowest scores as “low”, and the rest (40%) as “intermediate”. Consequently, the cutoff values were 0.269/0.887 for DFS and 3.435/4.344 for OS in CRC. K-M curves were applied to compare the survival differences. The K-M curve analysis showed statistically significant differences among the different risk groups (*p* < 0.0001) (**Figures 5A, B**). In the subgroup analysis, it was found that the correlation between nomogram-based risk stratification and DFS/OS was significant both in the subsets of stage II (DFS: *p* = 0.0017; OS: *p* = 0.0014) (**Figures 5C, D**) and stage III (DFS: *p* = 0.0028; OS: *p* = 0.0031) (**Figures 5E, F**) patients. Therefore, the risk scores generated based on our prognostic models efficiently distinguished the prognosis of patients with stage II or III CRC.

Based on our prognostic models, some patients previously believed to have a high risk of relapse or death rate were found to be at low risk. The classification of patients with stage III CRC into the low-risk (T_1–3_N_1_) and high-risk (T_4_ or N_2_) groups is routinely used to guide the treatment of adjuvant FOLFOX (folinic acid–fluorouracil–oxaliplatin) or CAPOX (capecitabine–oxaliplatin) in clinical practice (22). Based on this classification, our cohort included 17 low-risk (T_1–3_N_1_) and 86 high-risk (T_4_ or N_2_) patients with stage III CRC. As the number of patients in the low-risk group was too low, we only analyzed the high-risk group using our prognostic models. The analysis showed that our nomogram could significantly identify a group of patients with good OS, but not DFS, within clinically high-risk stage III CRC (**Supplementary Figures S4A, B**). Furthermore, we performed a similar analysis in patients with stage II CRC. High-risk stage II CRC had the characteristics of positive biomarkers for vascular infiltration, lymphatic infiltration, or nerve infiltration (VILINI^+^) or T_4_ stage II, whereas low-risk CRC was negative for VILINI markers (VILINI^–^) and T_1–3_ stage II (22). Our cohort included two low-risk (VILINI^–^ and T_1-3_) and 149 high-risk (VILINI^+^ or T_4_) patients with stage II CRC. As the number of patients in the low-risk (VILINI^–^ and T_1–3_) group was too low, we only analyzed the high-risk (VILINI^+^ or T_4_) group using our prognostic models. The analysis showed that our nomogram could significantly identify a group of patients with very good DFS and OS within clinically high-risk stage II CRC (**Supplementary Figures S4C, D**). These results suggest that our new multidimensional models could improve patient prognosis prediction.

**Figure 6:**
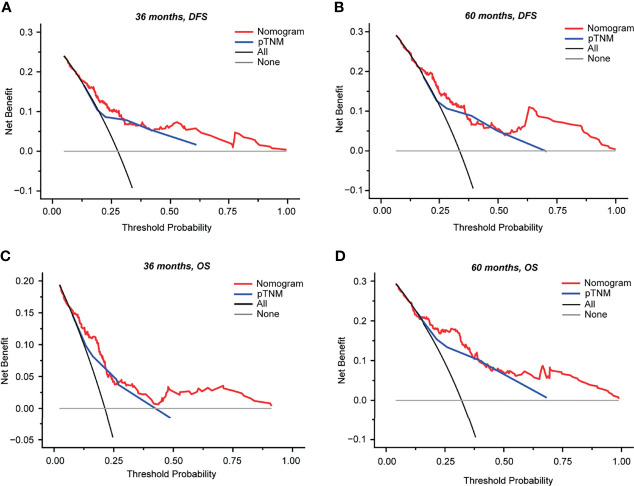
Decision curve analysis (DCA) of the nomograms and model comparisons with pTNM stage in 254 patients with stage II–III colorectal cancer (CRC). DCA was performed to determine the clinical usefulness of the nomogram *via* quantifying the net benefits at different threshold probabilities. The *x*-axis and *y*-axis represent the threshold probability and net benefit, respectively. **(A, B)** DCA curves for DFS at 36 months **(A)** and at 60 months **(B)**. **(C, D)** DCA curves for OS at 36 months **(C)** and at 60 months **(D)**. *Gray horizontal lines* denote that none of the patients received intervention. *Black lines* represent all patients who received intervention. *Red lines* represent the net benefit of model prediction. *Blue lines* represent the net benefit of pTNM prediction. DCA, decision curve analysis; OS, overall survival; DFS, disease-free survival; pTNM, pathological TNM based on the 8th edition of the American Joint Committee on Cancer (AJCC).

To determine the clinical usefulness of our nomograms, a decision curve analysis for the nomogram based on our model and pTNM stage was performed, as shown in **Figure 6**. By applying our prognostic models, a higher net benefit than that for the strategy of accepting or rejecting interventions for every patient could be achieved when the risk thresholds for DFS range from 12% to 100% at 3 years (**Figure 6A**) and from 16% to 100% at 5 years (**Figure 6B**) and for OS from 6% to 80% at 3 years (**Figure 6C**) and from 15% to 100% at 5 years (**Figure 6D**). Especially, at 3 years, if the threshold probability ranged from 0.12 to 0.30 and from 0.43 to 1.00, our prognostic models for DFS showed a better net benefit than that of pTNM stage (**Figure 6A**); at 5 years, if the threshold probability ranged from 0.16 to 0.36 and from 0.53 to 1.00, our prognostic models for DFS showed a better net benefit than that of pTNM stage (**Figure 6B**). As for the prognostic models for OS at 3 years, if the threshold probability ranged from 0.06 to 0.22 and from 0.27 to 0.80, our nomogram showed a better net benefit than that of pTNM stage (**Figure 6C**); at 5 years, our nomogram showed a better net benefit than that of pTNM stage if the threshold probability ranged from 0.15 to 0.37 and from 0.47 to 1.00 (**Figure 6D**).

### Website-Based Tool for Predicting the Prognosis of Stage II–III CRC

Based on the two prognostic models of the 254 patients from our cohort, we developed a website for predicting the risks of recurrence (DFS) and death (OS) of stage II–III CRC patients (http://www.biostatistics.online/liuyuan2). The scoring system based on our model was built into the website, and the prediction results, including risk stratification (high/intermediate/low) and recurrence/survival probabilities at different times, could be obtained after inputting the model variable values. It is easy to operate and friendly to clinicians, which is very helpful for the generalization and application of our models.

## Discussion

Postoperative recurrence and metastasis are the main factors affecting the survival of CRC patients after radical surgery. If the recurrence risk of CRC can be accurately predicted, more active interventions could be taken for high-risk patients and, therefore, they may have better survival benefits. Given the incomplete prognostic information of the present pTNM staging system, we integrated 18 variables including basic clinical indicators, preoperative serum tumor markers, blood inflammatory markers, MMR status, and the Immunoscore to generate prognostic models to evaluate the prognosis of stage II–III CRC. The model performance was validated and showed good accuracy and predictive ability. Furthermore, we developed a website based on our prognostic models, which is easy to use and of great convenience for the generalization and application of our models.

In this study, we validated the two-level categorical Immunoscore in patients with stage II–III CRC in our cohort whose prognostic impact was previously validated in stage I–III colon cancers (9). The MMR status was not a statistically significant factor for DFS/OS in our univariate analysis, and this could be attributed to the small sample size in our cohort (only 19 patients with MSI from the total 254 patients). In the chi-squared test analysis, we found that a high Immunoscore was more frequent than a low Immunoscore in tumors with dMMR. It is possible that the beneficial effect of a dMMR status for prognosis prediction could be attributed to its ability to induce strong antitumor immunity (which corresponds to a high Immunoscore) (18).

Recurrence risk stratification of patients is especially important for guiding clinicians to avoid both under- and overtreatment. Consequently, there is an urgent need to develop models that can accurately evaluate their prognosis and improve risk stratification management. This would guide patients regarding adjuvant chemotherapy and improve their treatment. The classification of patients with stage III CRC into low-risk (T_1–3_N_1_) and high-risk (T_4_ or N_2_) groups is routinely used to guide the treatment of adjuvant FOLFOX or CAPOX in clinical practice (22). As for stage II CRC patients, the NCCN and the European Society for Medical Oncology (ESMO) guidelines suggest that adjuvant chemotherapy may be considered in patients with high-risk features such as T4 staging, poor tumor differentiation, and the presence of lymphatic infiltration, vascular infiltration, or nerve infiltration (3, 23, 24). However, serum tumor markers, molecular characteristics, and the tumor immune microenvironment were not included in the determinants of chemotherapy decisions. Recently, a study by the Multicenter International Society for Immunotherapy of Cancer (SITC) of the consensus Immunoscore demonstrated the prediction of chemotherapy response in stage III colon cancer (25). This study showed that patients with a high Immunoscore significantly benefit from chemotherapy treatment, while patients with a low Immunoscore did not. Similarly, a randomized phase 3 clinical trial (IDEA) in 1,062 stage III colon cancer patients confirmed the predictive value of the Immunoscore on chemotherapy response (26). Therefore, the tumor immune microenvironment may play an important role in guiding the strategy for postoperative chemotherapy. Presently, the Sinicrope group and the Ghiringhelli group have developed comprehensive prognostic models including the Immunoscore for stage III colon cancer (18, 19). However, a comprehensive prognostic model containing the Immunoscore for stage II CRC still remains to be explored. Our cohort included 151 stage II CRC patients and 103 stage III CRC patients, so the prognostic models we developed can act as a good supplement to the models from the Sinicrope and Ghiringhelli groups for predicting stage II–III CRC outcomes.

Currently, there is no lack of research on early warning models for CRC recurrence (6, 7, 18, 19, 27, 28), but problems still exist. For example, patient data cannot include complete patient information and novel risk factors, or the indicators are not easy to obtain and of high cost, etc. Considering the clinical operation and cost, models with theoretically high predictive accuracy may not perform well in clinical practice, so it is not easy to achieve a balance between feasibility and accuracy. Our models have considerable advantages. Firstly, the abundant candidate variables provide relatively complete information for model construction, which helps to construct a model of high precision. Secondly, most of the variables included in this study, except for the Immunoscore (which can be obtained by immunohistochemistry with low cost and high consistency across different centers), are indicators of routine clinical tests, which are easy to obtain. Thirdly, we developed comprehensive prognostic models including the Immunoscore for stage II–III CRC, especially for stage II CRC. Fourthly, we built in a model scoring system to develop a model-based website, which greatly simplifies the scoring process and is therefore very user-friendly and beneficial to the generalization of our models. Finally, our prognostic models can make personalized patient predictions, which could contribute to the development of more precise medicine. Nevertheless, our models also have some shortcomings that need to be improved. Firstly, since this is a single-center retrospective study, it may produce selective bias and bring limitations regarding the generalizability of the model. Secondly, as our patients were collected from 2009 to 2016, at which time the *BRAF*/*KRAS*/*NRAS*/*HRAS* gene mutation state was not yet routinely screened for, our dataset lacked this information. Finally, due to the lack of an external dataset with matched sample type and variables, our models were only validated in the internal cohort. In our future work, we will validate our models in an independent external dataset. Furthermore, we will increase the number of research centers and sample size to expand the generalizability and application of our models.

In conclusion, we validated the two-level categorical Immunoscore in patients with stage II–III CRC. Furthermore, comprehensive models including clinicopathological indicators and the Immunoscore were constructed and validated, with good accuracy and convenience, to evaluate the risks of recurrence and death of stage II–III CRC patients. Our prognostic models may provide new insights into improving the current prognosis evaluation system and the quality of subsequent decision-making for postoperative follow-up and adjuvant treatment.

## Data Availability Statement

The raw data supporting the conclusions of this article will be made available by the authors, without undue reservation.

## Ethics Statement

The studies involving human participants were reviewed and approved by the Ethics Committee of Shanghai Jiao Tong University Affiliated Sixth People’s Hospital. The patients/participants provided written informed consent to participate in this study.

## Author Contributions

JR and SZ designed the study and interpreted the data. JR wrote the manuscript and collected the immunochemistry data and the related quantification data. ZW and DP amended the manuscript. WY, LY, and NS collected the clinicopathological and follow-up data from Shanghai Jiao Tong University Affiliated Sixth People’s Hospital. WY collected and stored the formalin-fixed paraffin-embedded sections for CD3^+^ and CD8^+^ T-cell staining. LFX, JO, and LX performed model construction and validation using the R language. LFX developed the website for predicting the prognosis of CRC. All authors contributed to the article and approved the submitted version.

## Funding

This work was supported by the Shanghai Municipal Education Commission-Gaofeng Clinical Medicine Grant Support (no. 20172023), China Postdoctoral Science Foundation (no. 2021M692121), National Natural Science Foundation of China (no. 82101833 and no. 31870829), Shanghai Municipal Health Commission, and the Collaborative Innovation Cluster Project (no. 2019CXJQ02).

## Conflict of Interest

The authors declare that the research was conducted in the absence of any commercial or financial relationships that could be construed as a potential conflict of interest.

## Publisher’s Note

All claims expressed in this article are solely those of the authors and do not necessarily represent those of their affiliated organizations, or those of the publisher, the editors and the reviewers. Any product that may be evaluated in this article, or claim that may be made by its manufacturer, is not guaranteed or endorsed by the publisher.

## Supplementary Material

Supplementary Figure 1 Study design.

Supplementary Figure 2 Validation of prognostic value of the two-level categorical Immunoscore in 254 patients. The Kaplan-Meier (K-M) method was applied to estimate DFS **(A)** and OS **(B)** probabilities at different times, and the log-rank test was performed to determine statistical differences. A 0–70% percentile Immunoscore was considered as low-Immunoscore, and a 70%-100% was considered as high-Immunoscore. K-M, Kaplan-Meier; OS, overall survival; DFS, disease-free survival.

Supplementary Figure 3 Predictive accuracy on DFS/OS of Immunoscore and other clinicopathological variables, or combined variables in 254 patients with stage II-III CRC. The integrated area under the ROC curve (iAUC) with 1000 × bootstrap resampling was used to evaluate the predictive accuracy on DFS **(A)** and OS **(B)** of different variables. Likelihood ratio tests were used for model performance comparison when the models were nested. OS, overall survival; DFS, disease-free survival; VILINI, vascular infiltration, lymphatic infiltration and nerve infiltration; CSA, tumor cross-sectional area; NLR, neutrophil-to-lymphocyte ratio; PLR, platelet-to-lymphocyte ratio; MMR, mismatch repair. *P < 0.05, **P < 0.01, ***P < 0.001.

Supplementary Figure 4 The nomograms improved patient prognosis prediction in clinical high-risk patients with stage II and stage III. **(A)** K-M DFS curve based on our nomogram in clinical high-risk stage III CRC patients (T4 or N2, n=86). **(B)** K-M OS curve based on our nomogram in high-risk stage III CRC patients (T4 or N2, n=86). **(C)** K-M DFS curve based on our nomogram in high-risk stage II CRC patients (VILINI+ or T4, n=149). **(D)** K-M OS curve based on our nomogram in high-risk stage II CRC patients (VILINI+ or T4, n=149). The log-rank test was performed to determine statistical differences. K-M, Kaplan-Meier; VILINI, vascular infiltration, lymphatic infiltration and nerve infiltration. OS, overall survival; DFS, disease-free survival.

## Abbreviations

AJCC, American Joint Committee on Cancer; CT, the core of the tumor; CSA, tumor cross-sectional area; C-index, concordance index; CIs, confidence intervals; CRC, colorectal cancer; CA19-9, carbohydrate antigen 19-9; CEA, carcinoembryonic antigen; CA125, carbohydrate antigen 125; DFS, disease-free survival; HR, hazard ratio; IM, invasive margin; iAUC, integrated area under the ROC curve; K-M, Kaplan–Meier; MSI, microsatellite instability; MSS, microsatellite stable; MMR, mismatch repair; dMMR, deficient mismatch repair; pMMR, proficient mismatch repair; NLR, neutrophil-to-lymphocyte ratio; OS, overall survival; PLR, platelet-to-lymphocyte ratio; pTNM, pathological TNM based on the 8th edition of AJCC; ROC, receiver operating characteristic; SITC, Society for Immunotherapy of Cancer; VILINI, vascular infiltration, lymphatic infiltration and nerve infiltration.
