# Supplementary figures and images for: Clinicopathological Features Combined With Immune Infiltration Could Well Distinguish Outcomes in Stage II and Stage III Colorectal Cancer: A Retrospective Study
Source: Front Oncol. 2021 Dec 3;11:776997. doi: 10.3389/fonc.2021.776997 (PMC8678133; doi:10.3389/fonc.2021.776997)

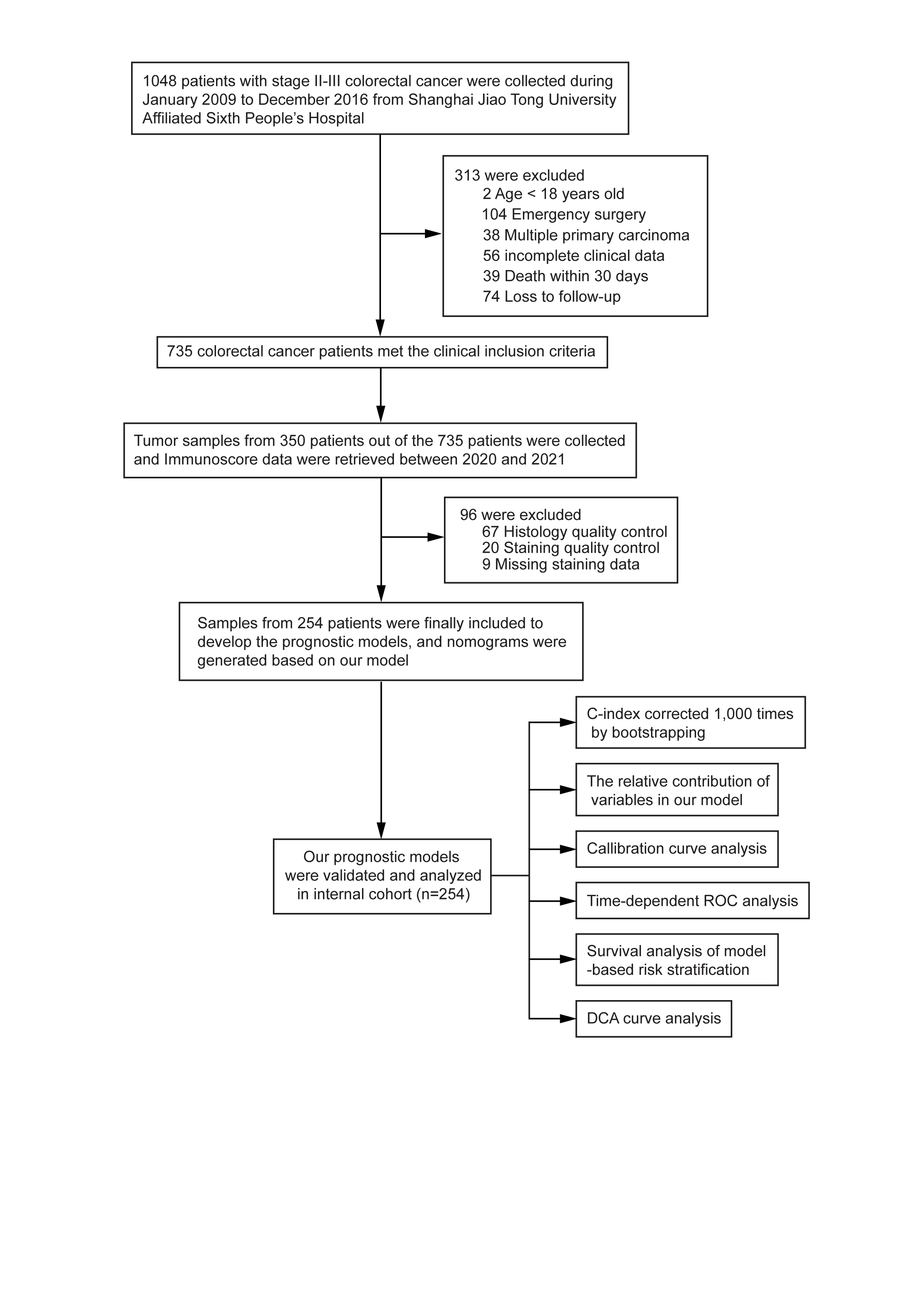

Supplement: Supplementary Figure 1 — Study design. [file Image_1.tif]

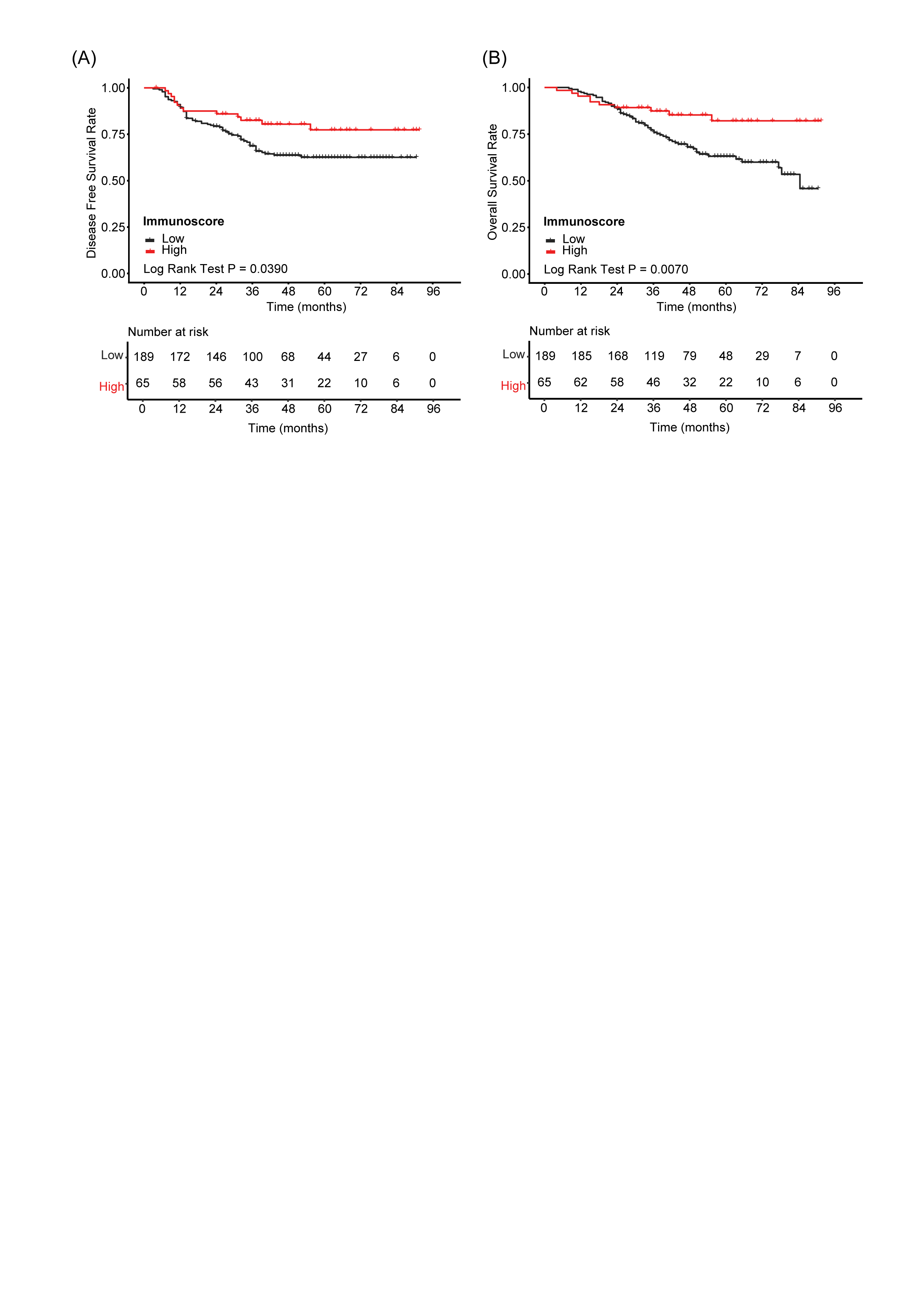

Supplement: Supplementary Figure 2 — Validation of prognostic value of the two-level categorical Immunoscore in 254 patients. The Kaplan-Meier (K-M) method was applied to estimate DFS (A) and OS (B) probabilities at different times, and the log-rank test was performed to determine statistical differences. A 0–70% percentile Immunoscore was considered as low-Immunoscore, and a 70%-100% was considered as high-Immunoscore. K-M, Kaplan-Meier; OS, overall survival; DFS, disease-free survival. [file Image_2.tif]

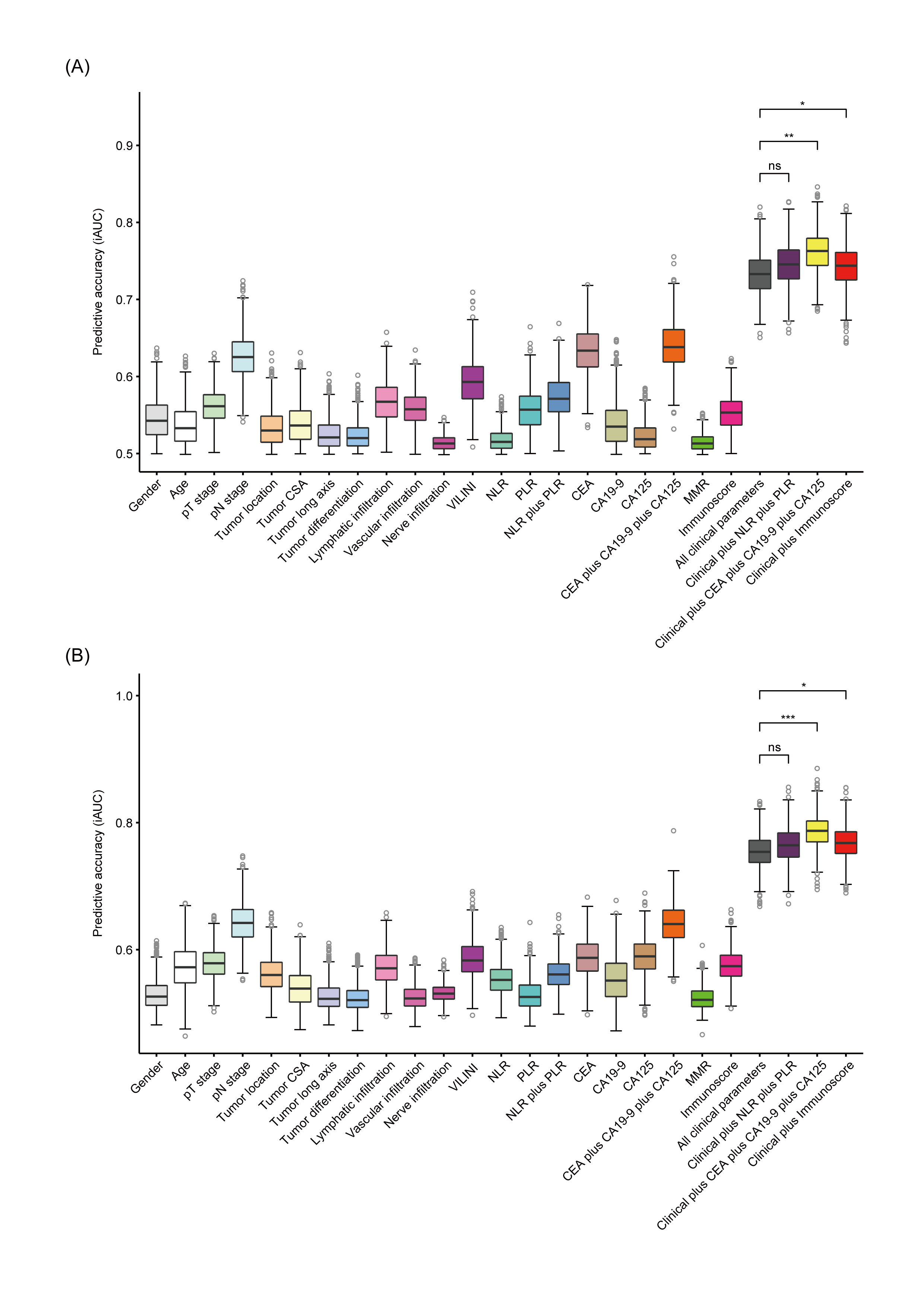

Supplement: Supplementary Figure 3 — Predictive accuracy on DFS/OS of Immunoscore and other clinicopathological variables, or combined variables in 254 patients with stage II-III CRC. The integrated area under the ROC curve (iAUC) with 1000 × bootstrap resampling was used to evaluate the predictive accuracy on DFS (A) and OS (B) of different variables. Likelihood ratio tests were used for model performance comparison when the models were nested. OS, overall survival; DFS, disease-free survival; VILINI, vascular infiltration, lymphatic infiltration and nerve infiltration; CSA, tumor cross-sectional area; NLR, neutrophil-to-lymphocyte ratio; PLR, platelet-to-lymphocyte ratio; MMR, mismatch repair. *P < 0.05, **P < 0.01, ***P < 0.001. [file Image_3.tif]

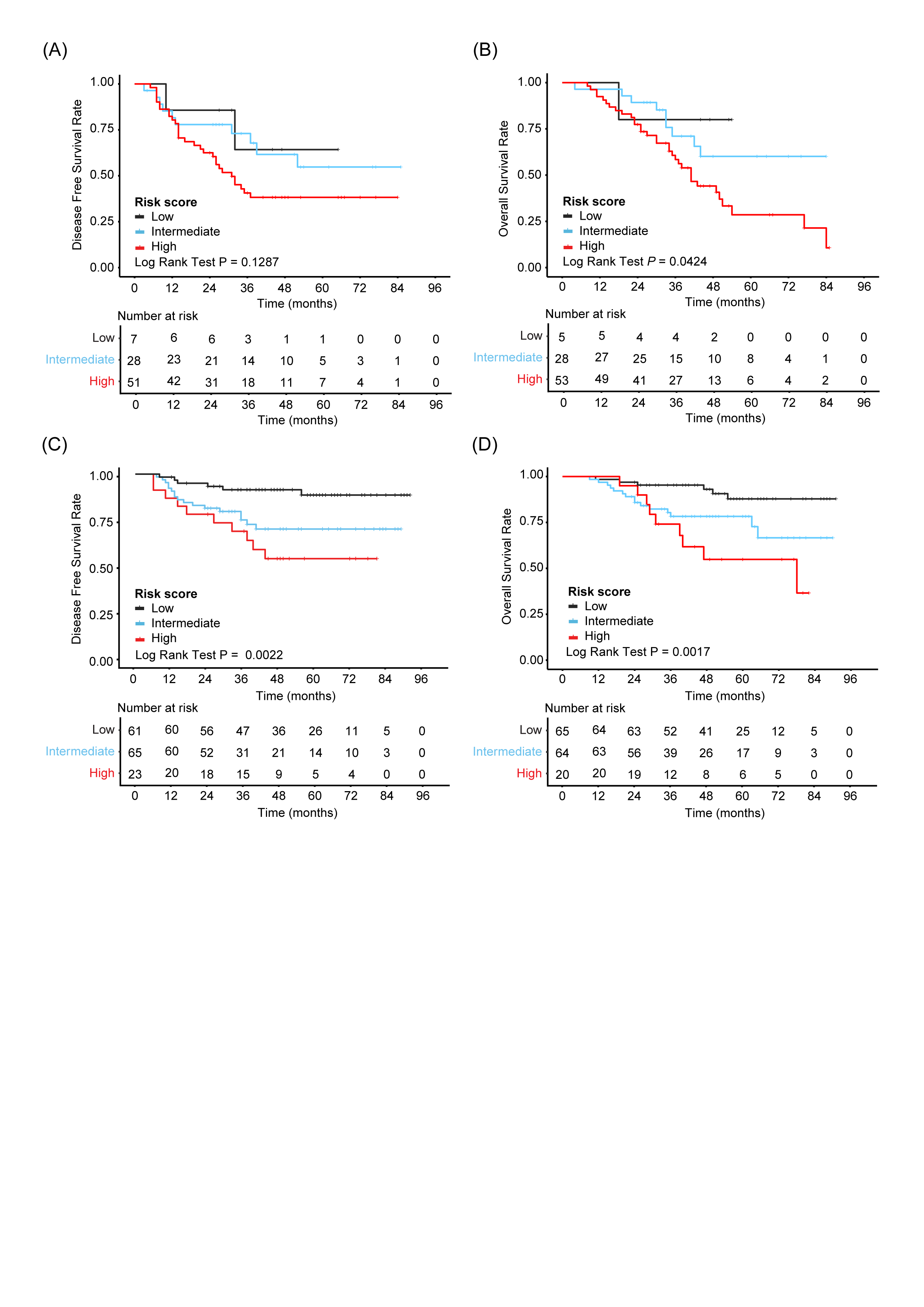

Supplement: Supplementary Figure 4 — The nomograms improved patient prognosis prediction in clinical high-risk patients with stage II and stage III. (A) K-M DFS curve based on our nomogram in clinical high-risk stage III CRC patients (T4 or N2, n=86). (B) K-M OS curve based on our nomogram in high-risk stage III CRC patients (T4 or N2, n=86). (C) K-M DFS curve based on our nomogram in high-risk stage II CRC patients (VILINI+ or T4, n=149). (D) K-M OS curve based on our nomogram in high-risk stage II CRC patients (VILINI+ or T4, n=149). The log-rank test was performed to determine statistical differences. K-M, Kaplan-Meier; VILINI, vascular infiltration, lymphatic infiltration and nerve infiltration. OS, overall survival; DFS, disease-free survival. [file Image_4.tif]
