# Supplementary material for: Clinicopathological Features Combined With Immune Infiltration Could Well Distinguish Outcomes in Stage II and Stage III Colorectal Cancer: A Retrospective Study
Source: Front Oncol. 2021 Dec 3;11:776997. doi: 10.3389/fonc.2021.776997 (PMC8678133; doi:10.3389/fonc.2021.776997)
Supplement: Supplementary file 5 [file Table_1.docx]

**Supplementary Table 1.** Correlation of Immunoscore with clinicopathological molecular characteristics

| Variable | n = 254 | Immunoscore | | |
| --- | --- | --- | --- | --- |
|  |  | Low | High | P value |
| Gender |  |  |  | 0.1716 |
| Female | 101 | 70 (37.0%) | 31 (48.0%) |  |
| Male | 153 | 119 (63.0%) | 34 (52.0%) |  |
| Age |  |  |  | 0.3579 |
| <65 | 116 | 90 (48.0%) | 26 (40.0%) |  |
| ≥65 | 138 | 99 (52.0%) | 39 (60.0%) |  |
| pT stage |  |  |  | 0.3307 |
| T1/T2/T3 | 61 | 42 (22.0%) | 19 (29.0%) |  |
| T4 | 193 | 147 (78.0%) | 46 (71.0%) |  |
| pN stage |  |  |  | 0.6164 |
| N0 | 151 | 109 (58.0%) | 42 (65.0%) |  |
| N1 | 67 | 52 (28.0%) | 15 (23.0%) |  |
| N2 | 36 | 28 (15.0%) | 8 (12.0%) |  |
| TNM stage |  |  |  | 0.4026 |
| II | 151 | 109 (58.0%) | 42 (65.0%) |  |
| III | 103 | 80 (42.0%) | 23 (35.0%) |  |
| Tumor location |  |  |  | 0.0689 |
| Left colon | 73 | 52 (28.0%) | 21 (32.0%) |  |
| Right colon | 84 | 70 (37.0%) | 14 (22.0%) |  |
| Rectum | 97 | 67 (35.0%) | 30 (46.0%) |  |
| Tumor CSA |  |  |  | 0.3918 |
| <16 | 123 | 95 (50.0%) | 28 (43.0%) |  |
| ≥16 | 131 | 94 (50.0%) | 37 (57.0%) |  |
| Long axis |  |  |  | 0.1323 |
| <4.5 | 124 | 98 (52.0%) | 26 (40.0%) |  |
| ≥4.5 | 130 | 91 (48.0%) | 39 (60.0%) |  |
| Degree of differentiation |  |  |  | 0.0748 |
| Moderate and well | 147 | 116 (61.0%) | 31 (48.0%) |  |
| Poor | 107 | 73 (39.0%) | 34 (52.0%) |  |
| Lymphatic infiltration |  |  |  | 0.1573 |
| Absent | 108 | 75 (40.0%) | 33 (51.0%) |  |
| Present | 146 | 114 (60.0%) | 32 (49.0%) |  |
| Vascular infiltration |  |  |  | 0.9371 |
| Absent | 224 | 166 (88.0%) | 58 (89.0%) |  |
| Present | 30 | 23 (12.0%) | 7 (11.0%) |  |
| Nerve infiltration |  |  |  | 0.9585 |
| Absent | 14 | 11 (6.0%) | 3 (5.0%) |  |
| Present | 240 | 178 (94.0%) | 62 (95.0%) |  |
| NLR |  |  |  | 0.4642 |
| Low | 222 | 163 (86.0%) | 59 (91.0%) |  |
| High | 32 | 26 (14.0%) | 6 (9.0%) |  |
| PLR |  |  |  | 1.0000 |
| Low | 216 | 161 (85.0%) | 55 (85.0%) |  |
| High | 38 | 28 (15.0%) | 10 (15.0%) |  |
| CEA |  |  |  | 0.8541 |
| Normal | 149 | 112 (59.0%) | 37 (57.0%) |  |
| Elevateed | 105 | 77 (41.0%) | 28 (43.0%) |  |
| CA19-9 |  |  |  | 0.2784 |
| Normal | 201 | 146 (77.0%) | 55 (85.0%) |  |
| Elevateed | 53 | 43 (23.0%) | 10 (15.0%) |  |
| CA125 |  |  |  | 0.7524 |
| Normal | 230 | 170 (90.0%) | 60 (92.0%) |  |
| Elevateed | 24 | 19 (10.0%) | 5 (8.0%) |  |
| MMR |  |  |  | 0.0468 |
| dMMR | 19 | 10 (5.0%) | 9 (14.0%) |  |
| pMMR | 235 | 179 (95.0%) | 56 (86.0%) |  |

Cutoff of NLR=5.17; cutoff of PLR=282.14; cutoff of CEA=5 ng/ml; cutoff of CA19-9=27 U/ml; cutoff of CA125=35 U/ml
