# Supplementary material for: Clinicopathological Features Combined With Immune Infiltration Could Well Distinguish Outcomes in Stage II and Stage III Colorectal Cancer: A Retrospective Study
Source: Front Oncol. 2021 Dec 3;11:776997. doi: 10.3389/fonc.2021.776997 (PMC8678133; doi:10.3389/fonc.2021.776997)
Supplement: Supplementary file 6 [file Table_2.docx]

**Supplementary Table 2.** Univariate Cox regression analyses for OS and DFS in patients with stage II-III CRC

|  | Disease-free survival | |  | Overall survival | |
| --- | --- | --- | --- | --- | --- |
| Variable | HR (95% CI) | P value |  | HR (95% CI) | P value |
| Gender |  |  |  |  |  |
| Male vs female | 1.42 (0.88-2.29) | 0.1504 |  | 1.21 (0.75-1.94) | 0.4369 |
| Age | 1.01 (0.99-1.03) | 0.3407 |  | 1.02 (1.00-1.04) | 0.0294 |
| pT stage |  |  |  |  |  |
| T4 vs T1/T2/T3 | 2.05 (1.11-3.80) | 0.0226 |  | 2.58 (1.32-5.04) | 0.0054 |
| pN stage |  |  |  |  |  |
| N1 vs N0 | 1.45 (0.83-2.52) | 0.1880 |  | 1.77 (1.01-3.09) | 0.0461 |
| N2 vs N0 | 3.98 (2.34-6.78) | 0.0000 |  | 4.27 (2.47-7.37) | 0.0000 |
| Tumor location |  |  |  |  |  |
| Right vs left | 1.14 (0.87-1.50) | 0.3439 |  | 1.05 (0.80-1.39) | 0.7071 |
| Degree of differentiation |  |  |  |  |  |
| Poor vs moderate and well | 1.01 (0.64-1.59) | 0.9669 |  | 1.10 (0.70-1.74) | 0.6768 |
| Tumor CSA | 0.99 (0.98-1.01) | 0.2525 |  | 0.99 (0.98-1.01) | 0.2964 |
| Tumor long axis | 0.99 (0.88-1.12) | 0.9254 |  | 1.00 (0.88-1.13) | 0.9681 |
| Lymphatic infiltration | 1.73 (1.07-2.78) | 0.0247 |  | 1.81 (1.10-2.96) | 0.0189 |
| Present vs absent |  |  |  |  |  |
| Vascular infiltration | 2.48 (1.43-4.31) | 0.0012 |  | 1.51 (0.80-2.87) | 0.2072 |
| Present vs absent |  |  |  |  |  |
| Nerve infiltration | 1.63 (0.52-5.19) | 0.4041 |  | 5.13 (0.71-36.97) | 0.1044 |
| Present vs absent |  |  |  |  |  |
| NLR | 1.00 (0.95-1.05) | 0.9865 |  | 1.05 (1.01-1.09) | 0.0106 |
| PLR | 1.00 (1.00-1.00) | 0.0866 |  | 1.00 (1.00-1.00) | 0.4684 |
| CEA | 1.46 (1.25-1.71) | 0.0000 |  | 1.30 (1.09-1.54) | 0.0028 |
| CA19-9 | 1.09 (0.90-1.32) | 0.3588 |  | 1.16 (0.95-1.41) | 0.1419 |
| CA125 | 0.99 (0.71-1.37) | 0.9496 |  | 1.55 (1.17-2.04) | 0.0020 |
| MMR |  |  |  |  |  |
| pMMR vs dMMR | 1.30 (0.52-3.21) | 0.5753 |  | 1.69 (0.62-4.64) | 0.3085 |
| Immunoscore |  |  |  |  |  |
| High vs low | 0.54 (0.30-0.98) | 0.0421 |  | 0.41 (0.21-0.80) | 0.0092 |
| Percentile Immunoscore | 0.34 (0.14-0.85) | 0.0214 |  | 0.33 (0.14-0.81) | 0.0159 |
| Three-level categorical Immunoscore | 0.65 (0.46-0.92) | 0.0141 |  | 0.70 (0.50-0.97) | 0.0321 |
| Intermediate vs low | 0.90 (0.51-1.57) | 0.7053 |  | 0.82 (0.48-1.40) | 0.4654 |
| High vs low | 0.38 (0.17-0.83) | 0.0157 |  | 0.47 (0.23-0.94) | 0.0341 |

Gender, pT stage, pN stage, tumor location, tumor differentiation, lymphatic infiltration, vascular infiltration, nerve infiltration, MMR status, and Immunoscore were evaluated as categorical variables, while age, tumor CSA, tumor long axis, NLR, PLR, CEA, CA19-9, CA125 were evaluated as continuous variables. CEA, CA19-9, and CA125 are processed by logarithmic transformation (base e); CI=confidence interval; HR= hazard ratio; CSA=tumor cross-sectional area; NLR=neutrophil-to-lymphocyte ratio; PLR=platelet-to-lymphocyte ratio; dMMR=deficient mismath repair; pMMR=proficient mismath repair. Percentile Immunoscore and three-level categorical Immunoscore are described by Galon et al(9).
